# The roles and experiences of informal carers providing care to people with advanced cancer in Africa—A systematic review and critical interpretive analysis

**DOI:** 10.1371/journal.pgph.0001785

**Published:** 2023-04-07

**Authors:** Rutendo G. Gambe, Joseph Clark, Stephanie A. Meddick-Dyson, Blessing O. Ukoha-Kalu, Gertrude N. Nyaaba, Fliss E. M. Murtagh

**Affiliations:** Wolfson Palliative Care Research Centre, Hull York Medical School, University of Hull, Hull, United Kingdom; Transcultural Psychosocial Organization Nepal / KIST Medical College, NEPAL

## Abstract

There is an increasing prevalence of cancer in Africa with approximately 80% of cancers diagnosed at an advanced stage. High out-of-pocket healthcare costs and overstretched health systems lead to heavy reliance on informal carers for cancer care. This study aims to explore the roles and experiences of informal carers including the impact of cancer care on individuals and communities and support available for carers. We carried out a systematic review following PRISMA reporting guidelines and used critical interpretative synthesis to identify themes and develop an informal carers’ experience framework. We searched nine databases and screened 8,123 articles from which 31 studies were included in the review. Most studies were from Sub-Saharan Africa (29/31, 94%), particularly Uganda (9, 29%). Carers were mostly women, aged 30–40 years, and siblings, spouses, or children. Caring roles included care coordination, fundraising, and emotional support. Caring was time-consuming with some carers reporting 121 hours/week of caring, associated with the inability to pursue paid work and depression. Four themes demonstrated carers’ experiences: 1) intrapersonal factors: strong sense of familial obligation, and grappling with gender roles, 2) interpersonal factors: impact of a cancer diagnosis on households, changing social and sexual relationships, 3) community factors: navigating cultural norms on nature and location of care, and 4) health system influences: barriers to accessing healthcare services, and tensions between traditional and biomedical medicine. These themes aligned with Bronfenbrenner’s social ecological model which aided our development of a framework for understanding informal carers’ experiences’. Our review highlights multifaceted roles and experiences of informal carers in Africa, amidst cultural and community impacts. Carers experience a strong obligation and willingly undertake the role of carer, but at the expense of their social, economic, and psychological wellbeing. Support for carers, including flexible working hours/ carers’ allowance, should be incorporated as part of universal health coverage.

## Introduction

Cancer prevalence in Africa is on the rise with annual cancer cases modelled to increase from 1,108,301 in 2020 to 2,082,732 cases by 2040 [1]. An aging population, combined with a rise in behavioural risk factors such as smoking, extensive alcohol consumption and decreased physical activity are contributory factors to an increasing prevalence of non-communicable diseases (NCDs) including cancer [2–4]. Up to 80% of cancer diagnoses in sub-Saharan Africa (SSA) are made at an advanced disease stage, resulting in up to four times higher mortality rates in SSA compared with those in high-income countries for similar cancers [2, 5]. A combination of poor access to treatment facilities, and out-of-pocket healthcare costs create major patient barriers to adequate cancer diagnosis and treatment [3]. A cancer diagnosis also has far reaching psychosocial, and financial implications on the patient’s household, including close relatives and non-relatives [6]. Compounding these patient and household factors are health system challenges around limited screening and early detection, insufficient health infrastructure, limited cancer workforce, and insufficient palliative care services which result in limited professional support for cancer patients across their disease trajectory [3]. Consequently, there is a heavy reliance on informal carers to supplement health system challenges to supporting cancer patients.

Informal carers are people (relatives and non-relatives) supporting patients, and often providing unpaid assistance with any activities of daily living for chronically ill patients [7]. They have been described as “fellow sufferers,” “co-patients,” and “co-afflicted” due to how they are also affected by a cancer diagnosis [6, 8, 9]. Informal carers are not only involved in the care of patients at the diagnosis and treatment stages but at the end of life as well, due to the high variability of palliative care services in Africa. Of 48 African countries surveyed by the African Palliative Care Association (APCA), 12 countries reported no hospice and palliative care services with 75% of all palliative care services in Africa concentrated in Kenya, Nigeria, South Africa, Tanzania and Uganda [10]. Such significant deficiencies and barriers to professional cancer and palliative care services mean informal carers play critical roles in the delivery of cancer care and supplement formal cancer and palliative care gaps.

Providing cancer care is multifaceted and crosses all aspects of a carer’s physical, psychological, social, and economic life. Its impacts on carers, range from instilling a sense of purpose in people and enabling personal/ spiritual growth to a loss of identity, foregoing financial/ educational opportunities and mental and emotional distress [7]. Informal carers’ well-being is therefore closely linked to the quality of informal care that patients receive [8]. Shedding light on the experiences of carers enables us to identify and prioritise them for support in health policy planning and implementation to improve health outcomes for informal carers and cancer patients in Africa [11].

While the role of informal carers in the provision of palliative care in Africa has been described mostly within the context of HIV/ AIDS [12], the role of informal carers in the provision of care for cancer patients in Africa is not clearly elucidated. Understanding the experiences of informal carers is key to providing evidence to better support them and incorporate them as essential partners in the provision of cancer and palliative care. This systematic review seeks to synthesise current evidence on the experiences of informal cancer carers in Africa. It provides contextual evidence on the characteristics, roles and experiences, challenges and support available for informal carers providing care to people with advanced cancer. It also details the varied impacts of such care giving on patients, families, and communities.

## Materials and methods

We conducted a systematic review using standard systematic review techniques, and according to Preferred Reporting Items for Systematic Reviews and Meta-Analyses (PRISMA) guidance [S1 Checklist] [13]. We used Critical Interpretive Synthesis to analyse our results and identify themes. This systematic review was registered with PROSPERO (Registration ID: CRD42022304894).

**Fig 1:**
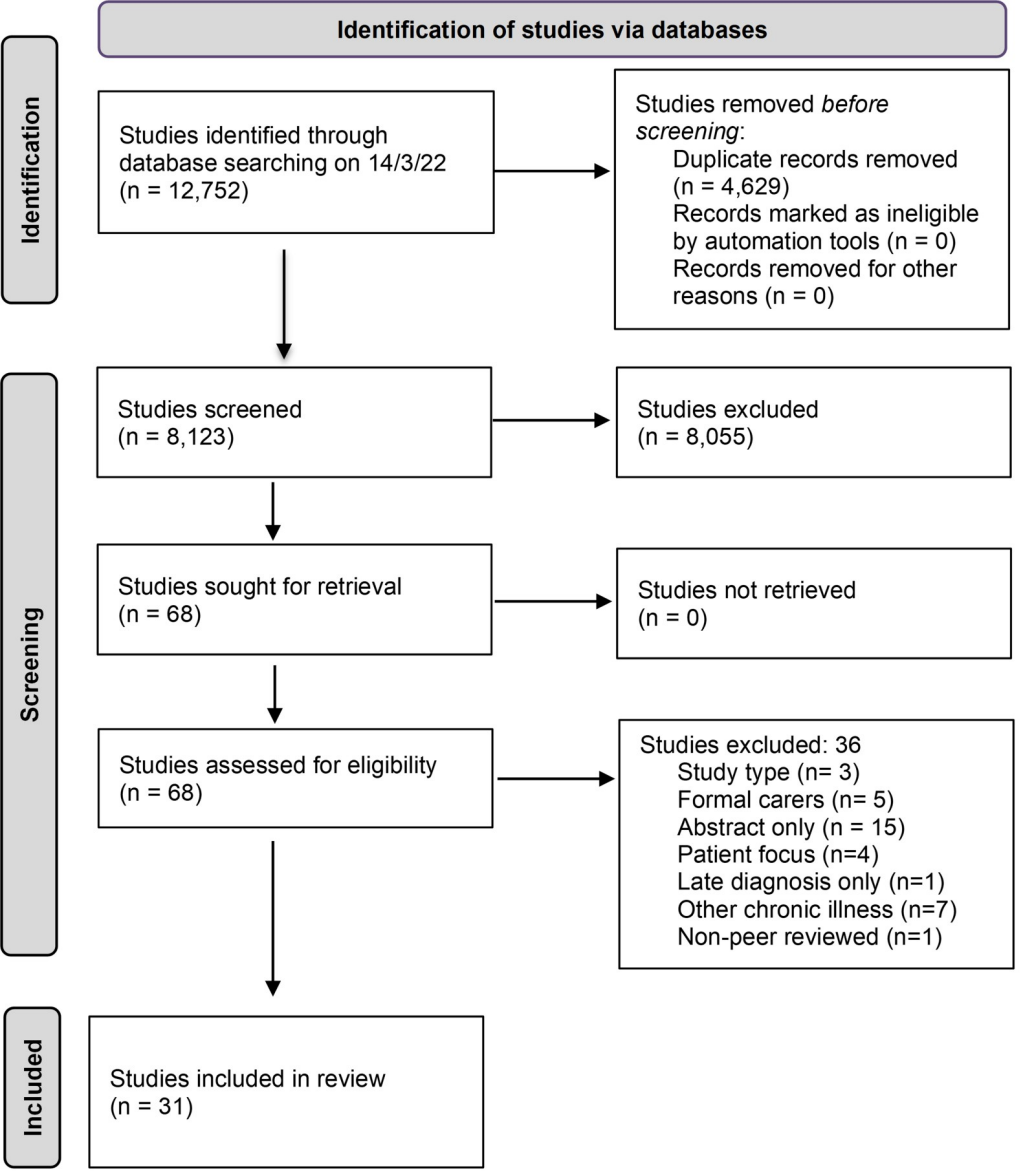
Preferred Reporting Items for Systematic Reviews and Meta-Analyses (PRISMA) flow chart.

Our search retrieved 8,123 studies after de-duplication. Sixty-eight studies were identified for full-text review and 31 studies were included for our final analysis (Fig 1).

### Search strategy and selection criteria

We searched nine databases (PubMed, EMBASE, Allied and Complementary Medicine Database (AMED), Web of Science, SCOPUS, PsycINFO, Cumulative Index of Nursing and Allied Health Literature (CINAHL), and African Journals Online) for articles published in 2000 and beyond, on 14 March 2022 using a search strategy outlined in S1 File. Search terms included i) synonyms for informal carers ii) cancer and its variations, and iii) Africa and each African country. These three parts were then combined by the ‘AND’ Boolean operator.

Inclusion and exclusion criteria are described in Table 1. We identified and included qualitative, quantitative, and mixed-methods research articles. We focused on adult informal carers providing care to a person with advanced cancer. We did not include literature pertaining to young carers (under 18), due to the distinct challenges they experience, such as balancing caring duties with, or leaving, education. We excluded literature focussing on people providing informal care to a person with cancer at earlier stages of illness due to most cancer diagnoses in SSA being made at an advanced stage [2].

**Table 1:** Inclusion and exclusion criteria.

Inclusion Criteria	Exclusion criteria
Literature reporting informal care of adults with advanced cancer	Studies conducted in countries/ regions outside Africa
Literature pertaining to countries in Africa/ regions of Africa	Studies addressing informal care in non-cancer patients
Qualitative, quantitative, mixed-methods research literature	Conference abstracts, non-peer-reviewed literature, grey literature, systematic reviews, case reports/series, editorial or commentaries
Published 2000-onwards	Studies addressing informal carers under 18 years of age
Studies published in English	
Studies pertaining to any cancer diagnosis	

### Study selection

Identified references were managed with Endnote20 [14] and Covidence systematic review management software [15]. All study titles and abstracts were screened for inclusion/ exclusion by two researchers (RG, SH) and a two other researchers (JC, FM) mediated any disagreements. Dual full-text screening was carried out on 20% of the screened titles and abstracts, with 98.5% agreement rate by two independent reviewers (RG, BUK) prior to quality appraisal and data extraction. Studies addressing informal carers of patients with diseases other than cancer were included if at least 50% of the patients being cared for had advanced cancer.

### Quality appraisal

Two researchers (RG and BUK) conducted quality appraisal using the Mixed Methods Appraisal Tool (MMAT) [16]. Any disagreements were resolved by consensus or with the involvement of a third reviewer (JC, FM). The MMAT is a validated tool, appropriate for the appraisal of qualitative, quantitative and mixed-method studies [16]. Instead of deriving an individual MMAT quality score for each study, the tool was instead used to highlight any methodological concerns. Given the limited number of studies within this area of work, we did not exclude any studies based on quality, but we report common methodological concerns from included studies to contextualise interpretation and analysis, and demonstrate a broad range of informal carer experiences.

### Data extraction

Data were extracted using a piloted data extraction form [S2 File]. We piloted our extraction form by extracting data from three studies to ensure that we captured fully the experiences and roles of informal carers (RG). This initial extraction was discussed between RG, JC and FM to ensure that extracted data were comprehensive and relevant to our research questions. In reading and re-reading the studies in the context, the extraction form was amended accordingly until the final version shown in S2 File was agreed upon as capturing the most details relevant to our review questions. Data extraction was performed by one researcher (RG), and 20% of the total studies were dually extracted for checking and comparison (GN) with high agreement [S2 File].

### Data analysis

Critical interpretive synthesis (CIS) is a useful method for synthesising qualitative and quantitative data to critically develop new theory [17]. Given that we were only able to include a limited number of studies from a wide range of countries, CIS was chosen as an appropriate method of synthesis to reflect the broad range of experiences of informal carers, highlight areas of common concern and develop a conceptual model, to situate the experiences of informal carers in context of families, communities and the healthcare system more broadly. We report our use of CIS to develop justifiable findings and conclusions transparently, but recognise that our use of a theory-driven approach to analysis of included studies means that that our findings are unlikely to be directly reproducible, even with full description of our analytical steps. To help us further outline key steps in our analysis process, we used the Enhancing transparency in reporting the synthesis of qualitative research (ENTREQ) checklist [S2 Checklist] [18].

The sequence of our analysis and synthesis was based on Dixon and Flemming’s approaches to CIS—1) reading and understanding each study in isolation, 2) identifying themes in the studies, and how these can be integrated into each other, 3) carrying out a reflective analysis to determine the significance and relevance of each theme, and their interactions, and 4) to develop a new conceptual framework of the components of an informal carers experience based on the evidence available [19, 20]. RG read all included papers in detail and extracted key information descriptively for discussion. Some papers did not focus primarily on the experiences of informal caregivers and only data relating to our research questions was extracted. Extracted data were organized in relation to discrete research questions relating to; roles and/ or experiences and/ or support [S2 File]. Two researchers (RG and JC) met regularly to review key findings from included studies and to identify relationships between key findings within included studies to develop initial themes, and sub-themes. Qualitative data most influenced the development of thematic areas and quantitative data were organized to support emergent themes. Emergent themes were then presented to the senior author (FM) who raised challenges about the organization of data and offered critical perspectives to draw out links between themes and highlight divergence. In re-reading and reflecting around data extracted in relation to emergent thematic areas (RG and JC), further developed themes relating to; the carers themselves (intrapersonal), widespread impact of cancer (interpersonal), community factors and healthcare systems. Themes were discussed and refined between RG, JC and FM until consensus was reached. These 4 themes were then used to describe the roles and experiences of informal carers, as well as the support available to them. Themes continued to develop during the writing process, in which some reorganization of themes was undertaken in order to most clearly present discrete findings within over-arching thematic areas. Quotes were selected between the research team which most clearly supported the issues raised within themes.

During the analytical process, we recognised significant overlap between aspects of each theme, indicating a close interplay between factors at individual, family, community and healthcare system levels. In order to highlight the inter-relatedness of experiences at each of these levels, we adapted Bronfenbrenner’s ecological framework which applies socioecological models to human development. Initially used to describe the elements (social networks, cultural and social attitudes around them) that feed into a child’s development, we adapted this framework to elucidate how components of informal carers’ experiences’ are influenced by the broader socio-ecological context in which informal care is provided [21, 22].

## Results

Of the 31 studies included in this review, 14 (45.2%) were qualitative, 15 (48.3%) were quantitative and two (6.5%) were mixed-methods studies. The qualitative studies used data collected using interviews and/or focus group discussions (12/14, 85.7%), and the photovoice method (2/14, 14.3%). All quantitative studies (15/15, 100%) were cross-sectional surveys (13/15, 86.7%) or questionnaires (2/15, 13.3%). The two mixed-method studies both combined interviews and surveys. Most studies were from SSA (n = 29), most of which were conducted in Uganda (n = 9) and Nigeria (n = 6) (Table 2). The two studies outside SSA were from Egypt and Morocco.

**Table 2:** Country representation of included studies (studies conducted in multiple countries were counted for each).

Country	Number of studies from country
Uganda	9
Nigeria	6
South Africa	4
Kenya	4
Ghana	4
Malawi	2
Egypt	1
Morocco	1
Togo	1

Included studies were generally methodologically sound when assessed using the MMAT for quality appraisal [S1 File]. Common issues identified were related to recruitment and sampling techniques used. Studies often recruited participants from single centres and used convenience sampling, which increased the risk of selection bias in the study populations. Nine studies measuring burden and mental health relied on patient self-reports which may have led to under- or overestimation of results.

Informal carers were predominantly women undertaking multiple and a diverse range of roles, including caring for family members or a loved one with a range of cancer diagnoses [Table 3, and S1, S2 Files].

**Table 3:** Characteristics and roles of informal carers.

**Who are informal carers?**	Women, aged between 30–40 *[7, 23–38]*, spouses, children, and siblings *[7, 23–26, 28, 30–49]*
**Who do they care for?**	Patients with cancers such as breast, cervical, colon, Kaposi’s sarcoma, oropharyngeal, lung, ovarian, uterine, leukemia, and prostate
**What are their roles?**	Assisted with activities of daily living–bathing, cooking, cleaning, assisting with toileting, laundry, overseeing hospital appointments (patient advocates), arranging doctors’ appointments, chaperoning, organising transport and paying for it, buying medications, decision makers, researchers and experimenters, care (wound care, catheter changes, stoma care, giving medications, adjusting doses, monitoring side effects), offering spiritual and emotional care for patients, navigating conversations around death and dying, fundraising, and financial support *[23, 30, 36, 42, 45–47, 49, 50]*

Caregiving was time consuming. 47.5% of 284 carers in a study conducted in Uganda spent more than 121 hours a week performing caring duties [35]. In another survey of 121 carers in Uganda, 32% provided between 6–12 hours of care per day, and 28% provided more than 12 hours of care a day [26]. In a Nigerian study, 30.6% of 157 informal carers spent more than 48 hours a week caring [39]. Carers often undertook the role without any professional training. In one study, 52% of 169 informal carers of patients under Hospice Uganda had received training on their caregiving roles [26].

### Identified themes

Using Critical Interpretive Synthesis, we identified four themes and 11 sub-themes (Table 4).

**Table 4:** Themes and subthemes.

Themes	Sub-themes
Intrapersonal/ individual factors: identity and evolving roles	• Gender identity, inequality, and familial obligation • Balancing new and old roles • Impact upon psychological wellbeing and quality of life • Deepening of spiritual and religious identity (coping)
Interpersonal factors: Cancer, the patient and beyond	• The widespread impact of a cancer diagnosis • Cancer type dependent challenges
Community factors and extended family: source of support and root for stigma	• Community and extended family as a blessing and curse • Navigating cultural protocols on who cares, and where caring takes place
Existing within a larger healthcare system	• The financial burden, and barriers to accessing services • Interaction with healthcare workers, and services • Role of traditional and complementary medical practitioners

### 1) Intrapersonal/ individual factors: identity and evolving roles

The carer role permeates into all personal areas of life of the carer. This theme captures challenges faced by individuals as they become informal carers—their motivations, internal challenges, and coping strategies.

#### Gender identity, inequality, and familial obligation

Undertaking the role of informal carer was commonly driven by a deep sense of obligation to the family and gendered expectations. Familial obligation was often framed around a cultural requirement, loyalty/setting as an example for younger family members, reciprocity/marital responsibility, and lacking any other choice [25, 30, 34, 47]. Several studies cited cultural viewpoints of caregiving as a woman’s role, although men undertake the role where a woman family member is not available [25, 29, 33, 47, 48].

“*When she was diagnosed of the disease (breast cancer) she had no one to take care of her, hence, as a brother, it is culturally expected that I take care of her. There are no women around, hence I have to do it” (Participant 3, brother) [47].*

The assumption that women will take on the role of carer limits women’s ability to pursue their own economic and educational aspirations, exacerbates poverty and renders them vulnerable to violence and exploitation [25, 30, 33]. In a study of carers in Malawi, women experienced decreased productivity measured by numbers of maize bags harvested, an increased experience of domestic violence, and pressures to marry for financial security [33]. Furthermore, despite women providing most informal care, this seldomly gave them decision making power within their communities or relationships. An informal carer in Kenya reported requiring permission from her husband to care for her brother in her marital home [30].

#### Balancing new and old roles

The role of carer affects carers’ identity, their social structure and ability to participate in social life. Carers described having to minimise how much time they spent outside the house, time with friends, and which community events they can attend and romantic relationships [34, 48]. Carers continuously balanced responsibilities such as parenting, being a spouse or taking care of parents with caring for the cancer patient [29, 49]. Other roles, such as employment and education were commonly foregone, often with reluctance and fear of the known implications of losing these roles [29].

“*Because of her situation I must always stay home, I cannot attend family meetings although compulsory for all family members; I don’t go to church anymore. I must always be with her to attend to her needs. I cannot take her out or allow others to visit because of her condition, least I attracts gossips and stigma. Her diagnosis has placed a lot of restrictions on my social life and now even some of family members and friends are angry with me”. (P009) [48]*

#### Impact upon psychological wellbeing and quality of life

Informal carers reported significant burden from providing care which negatively impacted their psychological wellbeing and quality of life. For instance, a study of 285 informal carers in Uganda found that 46.8% reported poor quality of life due to caring with the lowest quality of life reported amongst carers with low knowledge and self-perceived ability to support pain management [32]. In another Ugandan study of 284 carers, 35.2% were clinically depressed and 48.2% had clinical symptoms of anxiety [35]. Burden tended to increase with increased duration of caring, and low levels of education of the carer [32, 35, 39].

#### Deepening of spiritual and religious identity (coping)

Carers drew on religious beliefs for healing or sustenance [28, 42, 47, 48, 50]. Faith was sustaining in giving hope and understanding of current circumstances as demonstrated below:

“*I have a closer relationship with God than before. This is because devoid of God, I would not have had this strength. God is my pillar. I have faith in him as a healer and this calms me down with peace”. (P13) [48]*

Despite familial obligation and coping mechanisms, carers expressed an openness to accept external support. In a 2008 Ugandan study of 62 informal carers, 77% of carers expressed an openness to hire care if funds permitted [42].

### 2) Interpersonal factors: Cancer, the patient and beyond

This theme identified the impact of a cancer diagnosis beyond the patient.

#### The widespread impact of a cancer diagnosis

A cancer diagnosis extends beyond just being a medical diagnosis and impacts relationships and family dynamics. Carers spoke about navigating role reversal (from parents caring for children to children caring for parents) [30, 34], altered relationships (strengthening or fracturing of relationships) [25, 27] and dealing with a variety of emotions of their patient relative over course of the illness [25, 27, 34, 46]. Carers described “living with a changed person” [45] as changes in a patient’s personality and/or emotional state and the implications of these changes on the carer-patient dynamic post cancer diagnosis [25, 34, 45]. The strain on marital relations were frequently reported, including holding difficult conversations with patients, financial insecurity, and risk of marriage break-up due to high burden of care [30, 33, 48]:

“*He becomes short tempered… even when you talk to him nicely, he will answer you not in a nice way and he can even shout like you said he must be sick…I am trying to be strong for now [sniffs]…it hurts me…it is hard to live with a sick person, to financially support a sick person and my husband just gets mad for no reason. I even left my job so that I could just be home and look after him, but during this year it was hard” [45]*

Managing pain was one of the most distressing symptoms carers dealt with and was found to be associated with high emotional burden and was one of the largest predictors of anxiety and depression in carers [35, 37, 43, 45, 47].

*Hmm*, *the pain is very unbearable sometimes*. *The last time I cried*. *That made my wife and children also followed suit as if we were mourning*. [46]

#### Cancer-type dependent challenges

Different cancers present unique challenges to the patient and carer. In Malawi, 25% of carer and patient participants described sexual and physical violence in their relationships since a cancer diagnosis [33]. Cervical cancers were highlighted as causing sexual and physical violence for patients and their carers, and the threat of spousal abandonment [33]. For the cervical cancer patients, this included an inability to participate in sexual relationships with their partners, while for their carers, it meant being forced to use sex in exchange for food/ accommodation [33]. Breast cancers came with the challenge of sometimes needing to manage malignant wounds. Where this was not possible, and odour from the wound became an issue, carers and patients experienced further isolation and stigma from the community [29, 47]. Carers of prostate cancer patients described a heightened sense of familial obligation due to caring for a man/male who is considered as head of the family [46].

### 3) Community as a source of support and root for stigma

This theme captured the positive and negative roles that communities play in supporting patients and their carers through a cancer diagnosis.

#### Community and extended family as a blessing and curse

Many informal carers lived in multi-generational households, including all 103 participants in a study from Nigeria [40]. Extended family members provided support by: providing respite care, undertaking household tasks (cooking and cleaning), fundraising, providing transportation, aiding in making difficult decisions around end of life/ wills, being “courage givers”, providing financial support, listening ears, counselling [7, 25, 27, 29, 34, 44, 46–48, 50, 51]. Despite communities being reported as supportive, stigma and misperceptions related to a cancer diagnosis from community members were also present. Cancer sufferers could be perceived as infectious or contagious, ‘dead people walking’ or ‘be-witched’, and as people to avoid [48, 50, 51]. This isolated cancer patients and their carers and impacted help-seeking behaviours.

#### Navigating cultural protocols on who cares, and where caring takes place

In some studies, there were cultural expectations which presented challenges to carers and which limited their agency to provide care. A study of Kenyan women demonstrated the complexities of provision of personal care across genders, as female children are not supposed to see their fathers’ naked bodies, and vice versa [30]. In cases where an adult child was not present, younger, grandchildren were recruited to perform these tasks [30]. The study reported that both the parent and child experienced some degree of humiliation, shame and embarrassment when these cultural norms were not followed [30].

*I’m not supposed to see the nakedness of my father as a daughter. Yes. So now I had a lot of problems because ‘who will it do it for me?’ ‘Who will do it for me?’ [Silence] and my son is small–he’s 7years. He can’t do it for the grandfather*.

*But there was no choice. I had to cover him with a lesso [cotton cloth] then tell my son, ‘go remove for me the trousers, get this warm water and do like this’. I [gave] him some gloves … so he did it for me and then I took the clothes, washed [them], changed the bedding. [30]*

Furthermore, there was a delicate balance of where caring could take place, with, for example, married women unable to take care of their own families in the marital home [30].

### 4) Existing within a larger healthcare system

A lack of supportive services in many health care systems, meant that informal carers play a central role in all cancer care, in all settings. This theme explores how the presence and absence of health care infrastructure and policies impact the informal caregiving experience.

#### The significant financial burden, and barriers to accessing health care services

Financial strain was reported as a major source of stress, and burden among carers in most of the studies reviewed. Of the carers’ surveys in our studies, the range of financial strain ranged between 51% to 100% of informal carers experiencing significant financial strain [26, 27, 33, 42, 52, 53]. Cancer care costs are beyond the reach of the average household income, which can have prolonged, tragic effects on the family unit such as entrenching poverty. Families may need to move to be geographically closer to hospitals providing cancer care which can be destabilising for the family and can cause families to lose their home or necessitate children changing schools or dropping out school altogether [25, 33, 34, 46, 49, 54]:

“*The children, in fact, I have to withdraw the children from private school. They all going to public school just to in fact just to give myself little degree of mouth. (. . .) By 2016, I lost, we lost, we were not able to pay accommodation, we have to lodge for two years. The landlord take over, took possession of the house then we had to go to friends, my load as I speak is still spread outside. Then, err, a church member just allow her to stay at the balcony that’s where she has been. Rain she is there, sun she is there, night, mosquito everything, she is there. That way have tried to get accommodation, this January God provided a place for us, that we just move on the floor now, we are lying, just lie on the floor.” Male, 62, Nigeria” [49]*

Geographical distance to cancer healthcare services was reported as a key cause of financial strain and influences help-seeking behaviour. In South Africa, of the 286 patient/carer participants surveyed, 157 (55%) lived more than 50km from a cancer treatment centre [51]. Families are therefore forced to make judgements on the visits/ treatments/ medications they can forego due to geographical distance and travel costs in what can be termed as a ‘mix and match’ approach to medical care [34, 46, 49, 51]. In Togo, patients and carers describe rationalising their own treatment schedules based on what they can afford, rather than what is recommended by the medical teams, if they can seek any treatment [25].

“*Well, we’ve been given appointments so we could follow the appointments, but sometimes we fail to fulfil the appointments due to sometimes transport, and at times when we see there is really no necessity. if he has no…like, too much pain, or the condition is not bad, at times we find that we have to miss the appointment.” Male, 52, Uganda [49].*

#### Interaction with healthcare workers, and services

Where professional support was available, informal carers described the important help they received from all health care workers and how this alone was “life” giving. Participants describe the value of the counselling/education, medications, and care giving training they receive from healthcare facilities [46, 48, 50, 51, 54].

“*I like the way they attended to me when they realized I was not comfortable; they took time in the consulting room to counsel me and taught me how to clean and dress the wound at home using normal saline and home-made Metronidazole (Flagyl) cream. It would have been difficult to cope with the situation had it not been their support.” [48]*

Adequate continuity of care from a health professional was highlighted as supportive for carers. Informal carers described a strong desire to see the same healthcare professionals during each visit, requested for help with transport to the clinic, more flexibility in opening hours of clinics, being seen and acknowledged as part of the healthcare team, and more collaborative working with health professionals [25, 30, 34, 36, 48–50, 54].

#### Involvement of traditional and complementary medical practitioners

Informal carers expressed tensions between seeking help from traditional and complementary practitioners and biomedical health facilities (mostly hospitals). Traditional and complementary care was often used when the patient and carer participants felt they could not reach medical professionals or for illness conditions they felt were ‘unnatural’ or when it was cheaper to seek a traditional healer than go to the hospital [27, 46, 47, 50]. One participant in Ghana described a traditional method of caring for wounds which they found helpful [47]. Despite helpful advice with the management of some symptoms from traditional and complementary practitioners, other patients also reported negative experiences for symptoms such as urinary retention in prostate cancer care when hospitals were bypassed [46].

“*We went to an herbalist who gave us a mixture of charcoal and clay to apply on the wound to help with the bleeding. It really helped with the bleeding and helped in removing the sloughs. So I use it for the dressing at home” (caregiver participant) [47]*

*We got some herbal medicines from a woman who is known to be an expert in that field. I went to (name withheld), and I got those medicines because they said when I use them, I wouldn’t need to have the catheter in place. I paid a lot. After some time, my problem became worse (had sores at the anus). I went there again, and still, my problem wasn’t solved. I had to stop and report to the hospital (Ofori, patient) [46]*

### A social-ecological framework of the components of an informal carer’s experience

**Fig 2:**
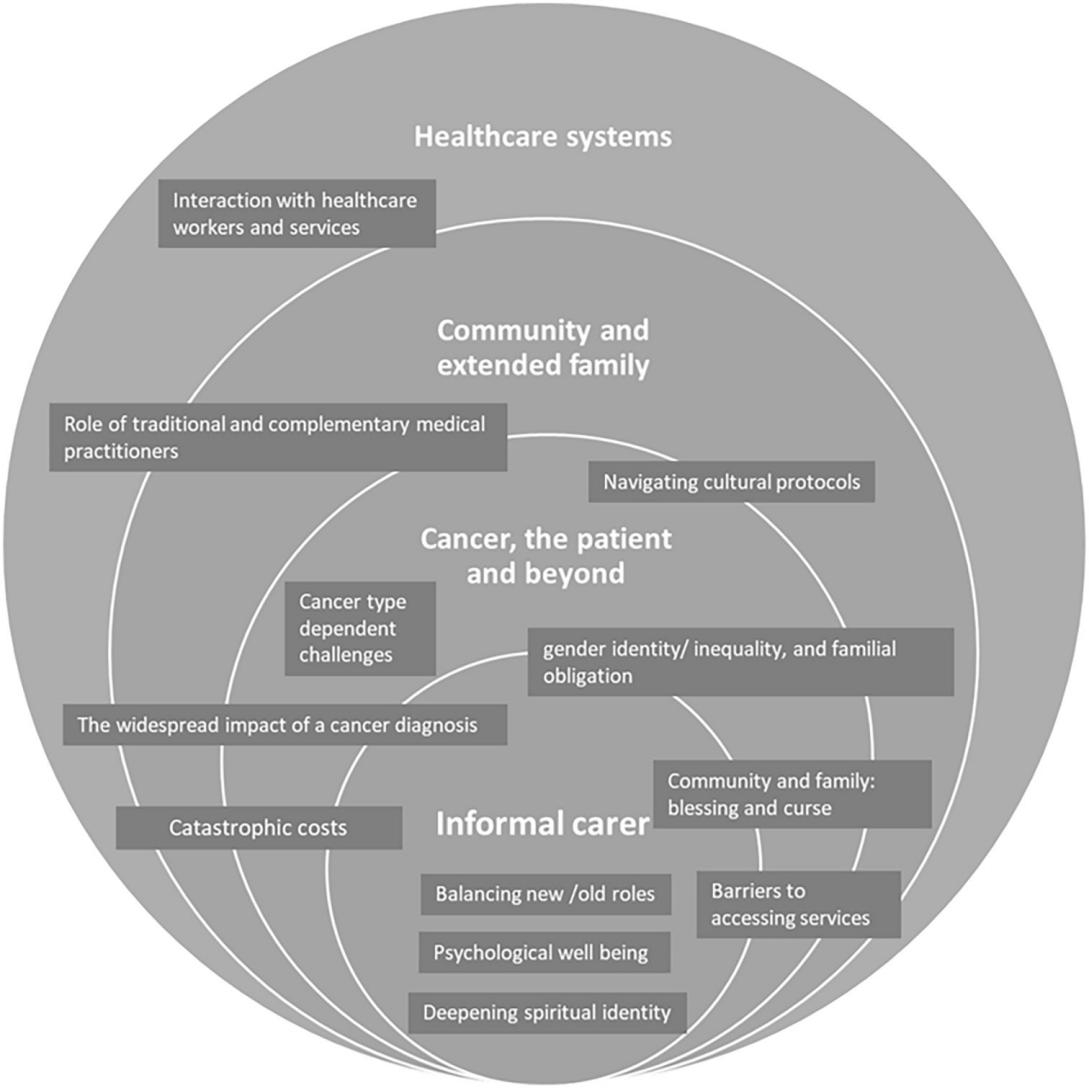
A social-ecological framework of the components of the informal carer’s experience (adapted from Bronfenbrenner’s social ecological model [21, 22]).

This review showed that the informal carer experience is impacted by four factors: 1) intrapersonal factors that are centred around the carer’s identity, 2) disease/ patient specific factors, 3) community and extended family and 4) health care systems in general. We adapted Bronfenbrenner’s social ecological model by placing the informal carer at the center of the model, and around it the factors that feed into their caregiving experience. The adapted framework showed how the sub-themes are intricately woven within and across individual themes, and impact different spheres of the informal carer’s experience (Fig 2).

## Discussion

Our review sought to understand the experiences of informal carers of cancer patients in Africa. We identified that informal carers’ experiences are complex, and impacted by individual, disease, familial and community and system-level dimensions. Interpreting themes using the social ecological model provides a framework for understanding carers’ experiences which places informal carers within a complex system of interactions with themselves, disease/patient, family, communities, and the wider health care system.

Efforts to improve cancer care in Africa commonly focus on infrastructure and healthcare workforce, education, availability of cancer treatments, and clinical research to improve models of cancer care. By doing so, the importance of informal carers and the circumstances in which they provide care around the patient are not given equal attention. Maree et al propose a family-centered approach to the care of cancer patients by identifying the layers that surround a cancer diagnosis at the patient/ family level [45]. Similarly, this review highlights the importance of assessing carer and patient needs independently of each other due to their unique challenges.

Informal carers across these 31 studies described the all-consuming nature of the role of caregiver, and the shift in identity and roles that comes alongside it. The transition into the role of informal carer, and the balancing of new and old roles is also described in other LMICs. In Iran, informal carers also describe the shift that came with assuming a role they are ill-prepared for, a requirement for self-sacrifice, and a sense of abandonment—the lack of support from both health workers and family and friends [55]. Similarly, informal carers of women with breast cancer in Mexico describe a ‘reorganisation’ of daily life which emphasises this idea of how life changes with a cancer diagnosis [56]. Across income-settings, there is a significant need for additional support for informal carers, in particular, educating carers with the practical skills necessary to provide informal care and support for the physical and emotional wellbeing for carers themselves.

Informal carers carry a significant part of the wider burden of care throughout the cancer disease trajectory–from diagnosis and treatment, through to end of life care. In a 2019 study, it was estimated that 27.3% of all deaths (uncertainty interval (UI) 25.2% - 29.6%) in high income countries occur in a person’s own home compared to 59.7% (UI 56.5%– 62.7%) in low-income and middle income countries and 79.5% (UI 77.3%– 81.5%) in low income countries [57]. These high proportions of home deaths echo our findings that high burdens of care lie outside formal healthcare and emphasise the centrality of the informal carer in providing care and support. Given the urgent need for measures to address health system challenges to providing adequate and accessible cancer and palliative care services, informal carers could be adequately resourced as key stakeholders for integrative cancer/ palliative care services at home.

A recurring theme from this review is the prevalence of stigma surrounding a cancer diagnosis, and how this serves to isolate families. These challenges are characteristic of some communities in Africa, but issues of cancer stigma have been reported in other parts of the world. In India for instance, women with breast and cervical cancer specifically reported isolation from communities and abuse due to stigma arising from fears of cancer being contagious, a form of punishment, and a death sentence [58–60]. This extended into subsequent worries about marriage eligibility or spreading the disease to their children [60]. Furthermore, these levels of stigma lead to altered health seeking behaviour and reduced the likelihood of disclosing illness to those around. These findings call for increased community education and awareness of cancer disease pathology to combat stigma around diagnosis and disease progression. Public health and media campaigns emphasising factually accurate information on what cancer is, how it spreads, and potential signs and symptoms, may be a helpful way of challenging stigma associated with cancer and could also promote appropriate health-seeking behaviour.

For consistent quality care for cancer patients, it is crucial to engage their carers, adequately support them and recognise them as critical partners in the provision of cancer care and end of life care. The all-consuming nature of informal care is not unique to African populations. In a 2017 post bereavement English survey, informal carers spent a median of 69 hours 30 minutes a week caring in the last 3 months of life [61], which correlates closely with findings in our studies. Consequently, a significant number of carers, described an inability to continue working when taking on an informal caregiving role. Introducing workplace policies to protect employment for people in informal caring roles such as flexible hours and paid/ partially paid leave/ carers’ allowance will be of benefit for people taking on the caring role. In a 2021 study, palliative care services were shown to limit financial toxicity and improve quality of life [62]. By improving access to palliative care services and ensuring that palliative care is part of universal health coverage, households can be protected from catastrophic costs.

Capacity to pay for care is a major deterrent to accessing cancer care in Africa. Whitehead et al described the concept of a ‘medical poverty trap’ where sickness can lead to poverty and/ or exacerbate the poverty some families are already in [63]. This poverty cycle is well demonstrated in the studies we reviewed with late presentation for cancer care, and the ‘mixing and matching’ that families are forced to do with regards to their medical care due to cost. The financial burden of cancer care remained a recurrent burden mentioned in our studies and resulted in significant variation in cancer outcomes within and between countries. Furthermore, catastrophic financial burdens are not unique to patients with cancer and their families in Africa alone. In a review of financial toxicity associated with cancer in LMICs, Donkor et al described a prevalence between 17.8% and 93.4% of financial toxicity amongst cancer patients [64]. These findings strongly point towards the need for support for carers being incorporated as part of universal health coverage to reduce out of pocket spending, and promote equity. In addition to this, governments can regulate cancer care costs, and cap prices on cancer, and supportive care medications to limit the financial burden on individuals.

Up to 80% of people in sub-Saharan Africa are estimated to seek advice and care from traditional healers [3]. Governments could facilitate and engage with discussion within communities about the role of traditional and complementary medicine practitioners to ensure that their roles are clearly outlined, and to establish traditional and complementary medicine practitioners as formal partners in managing symptoms of cancer.

## Conclusions

This systematic review highlights the many, and multifaceted roles and experiences of informal carers of advanced cancer patients in Africa. A diagnosis of cancer extends beyond the patient’s own physical, mental, spiritual, and financial wellbeing and significantly impacts their carers, families, and communities in all these domains. Carers are spurred on by familial obligation but also grapple with self-identity, gender roles and maintaining good mental health. Carers learn to negotiate between their caring role, and the expectations that families lay on them. Furthermore, carers contend with high costs of cancer care, are often forced to pick and choose care that is affordable or find other avenues of treatment and symptom management from traditional and alternative medical practitioners. Carers find themselves at the center of interactions between themselves, the patient and disease, communities, and the wider healthcare systems.

By looking at cancer care through the lens of the informal carer, and acknowledging them as key players in cancer care, patients with advanced cancer can ultimately be better supported, and some of the catastrophic implications of a cancer diagnosis on a household better contained. As informal carers are coordinators and managers of care, they are well placed to liaise with the patient, health care services and the community to maximise patient autonomy as well as the care received by advanced cancer patients.

### Strengths and limitations

A key strength of our study is that we included all studies of informal carers of a person with advanced cancer in Africa, allowing us to report in-depth and broad ranges of experiences of informal carers. Use of critical interpretive synthesis enabled us to integrate qualitative and quantitative data and allowed us to not only analyse the primary data but to synthesise and develop a new framework from the data. Though this presents itself as a strength of our analysis, the interpretive and reflective element of CIS makes it hard for our analysis to be reproducible. We have included all details of the information extracted and the processes used to reach our conclusions. Studies included in our review form a limited evidence-base which introduces several biases. Published research comes from only nine countries in Africa where palliative care services are known to be present. Participants in included studies were all identified in healthcare settings, which itself indicates a level of access to healthcare which is not available to the majority of people given poverty levels and geographical access. Findings therefore may not be generalisable.

## Supporting information

S1 Checklist “PRISMA checklist”. (DOCX)

S2 Checklist “ENTERQ checklist”. (DOCX)

S1 File “Search strategy and quality appraisal”. (XLSX)

S2 File “Data extraction form and details of included studies”. (DOCX)
