# Supplementary material for: The roles and experiences of informal carers providing care to people with advanced cancer in Africa—A systematic review and critical interpretive analysis
Source: PLOS Glob Public Health. 2023 Apr 7;3(4):e0001785. doi: 10.1371/journal.pgph.0001785 (PMC10081800; doi:10.1371/journal.pgph.0001785)
Supplement: S2 File — (DOCX) [file pgph.0001785.s004.docx]

**Supplementary information**

Search strategy for all databases

The following electronic databases were searched using a pre-defined search strategy: PubMed, EMBASE, Allied and Complementary Medicine Database (AMED), Web of Science, SCOPUS, PsycINFO, Cumulative Index of Nursing and Allied Health Literature (CINAHL), and African Journals online

PubMed

1. “Informal caregiver*” (MeSH terms) (Caregiver* OR Carer* OR “Care Giver*” OR Spouse OR “Spouse Caregiver*” OR Family OR “Family Caregiver*”)
2. “Advanced Cancer*” (MeSH terms) (Cancer* OR “Benign Neoplas*” OR Malignancy OR “Malignant Neoplasm*” OR “Metastatic Cancer*” OR Neoplas* OR Tumo* OR “terminal cancer*” OR “end stage cancer*” OR palliative OR hospice OR dying OR “end of life” OR “end-of-life” OR “life threatening” OR “life-threatening”)
3. “Africa” (MesH terms) (Southern Africa OR Central Africa OR East Africa OR West Africa OR North Africa OR African OR Algeria OR Angola OR Benin OR Botswana OR Burkina Faso OR Burundi OR Cameroon OR Cape Verde OR Central African Republic OR Chad OR Comoros OR Congo OR Cote d'Ivoire OR Egypt OR Equatorial Guinea OR Eritrea OR Eswatini OR Ethiopia OR Gabon OR Gambia OR Ghana OR Guinea OR Kenya OR Lesotho OR Liberia OR Libya OR Madagascar OR Malawi OR Mali OR Mauritania OR Mauritius OR Morocco OR Mozambique OR Namibia OR Niger OR Nigeria OR Rwanda OR Sao Tome OR Principe OR Senegal OR Seychelles OR Sierra Leone OR Somalia OR South Africa OR Sudan OR Sahrawi OR Swaziland OR Tanzania OR Togo OR Tunisia OR Uganda OR Zambia OR Zimbabwe)
4. post 2000
5. 1 AND 2 AND 3 AND 4

EMBASE

1. Informal caregiver* OR Caregiver* OR Carer* OR Care Giver* OR Spouse OR Spouse Caregiver* OR Family OR Family Caregiver*
2. Advanced Cancer* OR Cancer* OR Benign Neoplas* OR Malignancy OR Malignant Neoplasm* OR Metastatic Cancer* OR Neoplas* OR Tumo* OR terminal cancer* OR end stage cancer* OR palliative OR hospice OR dying OR end of life OR end-of-life OR life threatening OR life-threatening
3. Africa OR Southern Africa OR Central Africa OR East Africa OR West Africa OR North Africa OR African OR Algeria OR Angola OR Benin OR Botswana OR Burkina Faso OR Burundi OR Cameroon OR Cape Verde OR Central African Republic OR Chad OR Comoros OR Congo OR Cote d'Ivoire OR Egypt OR Equatorial Guinea OR Eritrea OR Eswatini OR Ethiopia OR Gabon OR Gambia OR Ghana OR Guinea OR Kenya OR Lesotho OR Liberia OR Libya OR Madagascar OR Malawi OR Mali OR Mauritania OR Mauritius OR Morocco OR Mozambique OR Namibia OR Niger OR Nigeria OR Rwanda OR Sao Tome OR Principe OR Senegal OR Seychelles OR Sierra Leone OR Somalia OR South Africa OR Sudan OR Sahrawi OR Swaziland OR Tanzania OR Togo OR Tunisia OR Uganda OR Zambia OR Zimbabwe
4. 1 AND 2 AND 3

Allied and Complementary Medicine Database (AMED)

1. Informal caregiver* OR Caregiver* OR Carer* OR Care Giver* OR Spouse OR Spouse Caregiver* OR Family OR Family Caregiver*
2. Advanced Cancer* OR Cancer* OR Benign Neoplas* OR Malignancy OR Malignant Neoplasm* OR Metastatic Cancer* OR Neoplas* OR Tumo* OR terminal cancer* OR end stage cancer* OR palliative OR hospice OR dying OR end of life OR end-of-life OR life threatening OR life-threatening
3. Africa OR Southern Africa OR Central Africa OR East Africa OR West Africa OR North Africa OR African OR Algeria OR Angola OR Benin OR Botswana OR Burkina Faso OR Burundi OR Cameroon OR Cape Verde OR Central African Republic OR Chad OR Comoros OR Congo OR Cote d'Ivoire OR Egypt OR Equatorial Guinea OR Eritrea OR Eswatini OR Ethiopia OR Gabon OR Gambia OR Ghana OR Guinea OR Kenya OR Lesotho OR Liberia OR Libya OR Madagascar OR Malawi OR Mali OR Mauritania OR Mauritius OR Morocco OR Mozambique OR Namibia OR Niger OR Nigeria OR Rwanda OR Sao Tome OR Principe OR Senegal OR Seychelles OR Sierra Leone OR Somalia OR South Africa OR Sudan OR Sahrawi OR Swaziland OR Tanzania OR Togo OR Tunisia OR Uganda OR Zambia OR Zimbabwe
4. 1 AND 2 AND 3

Web of Science

1. Informal caregiver* OR Caregiver* OR Carer* OR Care Giver* OR Spouse OR Spouse Caregiver* OR Family OR Family Caregiver*
2. Advanced Cancer* OR Cancer* OR Benign Neoplas* OR Malignancy OR Malignant Neoplasm* OR Metastatic Cancer* OR Neoplas* OR Tumo* OR terminal cancer* OR end stage cancer* OR palliative OR hospice OR dying OR end of life OR end-of-life OR life threatening OR life-threatening
3. Africa OR Southern Africa OR Central Africa OR East Africa OR West Africa OR North Africa OR African
4. 1 AND 2 AND 3

SCOPUS

1. Informal caregiver* OR Caregiver* OR Carer* OR Care Giver* OR Spouse OR Spouse Caregiver* OR Family OR Family Caregiver*
2. Advanced Cancer* OR Cancer* OR Benign Neoplas* OR Malignancy OR Malignant Neoplasm* OR Metastatic Cancer* OR Neoplas* OR Tumo* OR terminal cancer* OR end stage cancer* OR palliative OR hospice OR dying OR end of life OR end-of-life OR life threatening OR life-threatening
3. Africa OR Southern Africa OR Central Africa OR East Africa OR West Africa OR North Africa OR African OR Algeria OR Angola OR Benin OR Botswana OR Burkina Faso OR Burundi OR Cameroon OR Cape Verde OR Central African Republic OR Chad OR Comoros OR Congo OR Cote d'Ivoire OR Egypt OR Equatorial Guinea OR Eritrea OR Eswatini OR Ethiopia OR Gabon OR Gambia OR Ghana OR Guinea OR Kenya OR Lesotho OR Liberia OR Libya OR Madagascar OR Malawi OR Mali OR Mauritania OR Mauritius OR Morocco OR Mozambique OR Namibia OR Niger OR Nigeria OR Rwanda OR Sao Tome OR Principe OR Senegal OR Seychelles OR Sierra Leone OR Somalia OR South Africa OR Sudan OR Sahrawi OR Swaziland OR Tanzania OR Togo OR Tunisia OR Uganda OR Zambia OR Zimbabwe
4. 1 AND 2 AND 3

PsycINFO

1. Informal caregiver* OR Caregiver* OR Carer* OR Care Giver* OR Spouse OR Spouse Caregiver* OR Family OR Family Caregiver*
2. Advanced Cancer* OR Cancer* OR Benign Neoplas* OR Malignancy OR Malignant Neoplasm* OR Metastatic Cancer* OR Neoplas* OR Tumo* OR terminal cancer* OR end stage cancer* OR palliative OR hospice OR dying OR end of life OR end-of-life OR life threatening OR life-threatening
3. Africa OR Southern Africa OR Central Africa OR East Africa OR West Africa OR North Africa OR African OR Algeria OR Angola OR Benin OR Botswana OR Burkina Faso OR Burundi OR Cameroon OR Cape Verde OR Central African Republic OR Chad OR Comoros OR Congo OR Cote d'Ivoire OR Egypt OR Equatorial Guinea OR Eritrea OR Eswatini OR Ethiopia OR Gabon OR Gambia OR Ghana OR Guinea OR Kenya OR Lesotho OR Liberia OR Libya OR Madagascar OR Malawi OR Mali OR Mauritania OR Mauritius OR Morocco OR Mozambique OR Namibia OR Niger OR Nigeria OR Rwanda OR Sao Tome OR Principe OR Senegal OR Seychelles OR Sierra Leone OR Somalia OR South Africa OR Sudan OR Sahrawi OR Swaziland OR Tanzania OR Togo OR Tunisia OR Uganda OR Zambia OR Zimbabwe
4. 1 AND 2 AND 3

Cumulative Index of Nursing and Allied Health Literature (CINAHL)

S1. Informal caregiver* OR Caregiver* OR Carer* OR Care Giver* OR Spouse OR Spouse Caregiver* OR Family OR Family Caregiver*

S2. Advanced Cancer* OR Cancer* OR Benign Neoplas* OR Malignancy OR Malignant Neoplasm* OR Metastatic Cancer* OR Neoplas* OR Tumo* OR terminal cancer* OR end stage cancer* OR palliative OR hospice OR dying OR end of life OR end-of-life OR life threatening OR life-threatening

S3. Africa OR Southern Africa OR Central Africa OR East Africa OR West Africa OR North Africa OR African OR Algeria OR Angola OR Benin OR Botswana OR Burkina Faso OR Burundi OR Cameroon OR Cape Verde OR Central African Republic OR Chad OR Comoros OR Congo OR Cote d'Ivoire OR Egypt OR Equatorial Guinea OR Eritrea OR Eswatini OR Ethiopia OR Gabon OR Gambia OR Ghana OR Guinea OR Kenya OR Lesotho OR Liberia OR Libya OR Madagascar OR Malawi OR Mali OR Mauritania OR Mauritius OR Morocco OR Mozambique OR Namibia OR Niger OR Nigeria OR Rwanda OR Sao Tome OR Principe OR Senegal OR Seychelles OR Sierra Leone OR Somalia OR South Africa OR Sudan OR Sahrawi OR Swaziland OR Tanzania OR Togo OR Tunisia OR Uganda OR Zambia OR Zimbabwe

S4. 1 AND 2 AND 3

African Journals online (terms searched as individual terms)

1. Informal caregiver
2. Carer
3. Spouse
4. Family
5. Advanced Cancer
6. Cancer
7. Palliative
8. Hospice
9. End of life
10. Africa
11. Southern Africa
12. Central Africa
13. East Africa
14. West Africa
15. North Africa
16. African

**Table S1**: Quality appraisal using the MMAT

**Qualitative**

| **Study** | **S1. Are there clear research questions?** | **S2. Do the collected data allow to address the research questions?** | 1.1. Is the qualitative approach appropriate to answer the research question? | 1.2. Are the qualitative data collection methods adequate to address the research question? | 1.3. Are the findings adequately derived from the data? | 1.4. Is the interpretation of results sufficiently substantiated by data? | 1.5. Is there coherence between qualitative data sources, collection, analysis and interpretation? |
| --- | --- | --- | --- | --- | --- | --- | --- |
| Adejoh, 2021 | yes | yes | yes | yes | yes | yes | yes |
| Mensah 2021 | yes | yes | yes | yes | yes | yes | yes |
| Kusi 2020 | yes | yes | yes | yes | yes | yes | yes |
| Salifu 2020 | yes | yes | yes | yes | yes | yes | yes |
| Bessa 2012 | yes | yes | yes | yes | yes | yes | yes |
| Maree 2017 | yes | yes | yes | yes | yes | yes | yes |
| Githaiga 2016 | yes | yes | yes | yes | yes | yes | yes |
| Githaiga 2015 | yes | yes | yes | yes | yes | yes | yes |
| Mlaba 2021 | yes | yes | yes | yes | yes | yes | yes |
| Mkandawire-Valhmu, 2020 | yes | yes | yes | yes | yes | yes | yes |
| Githaiga 2017 | yes | yes | yes | yes | yes | yes | yes |
| Potgieter, 2018 | yes | yes | yes | yes | yes | yes | yes |
| Bates 2018 | yes | yes | yes | yes | yes | yes | yes |
| Edwards 2018 | yes | yes | yes | yes | yes | yes | yes |

**Quantitative**

| Study | **S1. Are there clear research questions?** | **S2. Do the collected data allow to address the research questions?** | 4.1. Is the sampling strategy relevant to address the research question? | 4.2. Is the sample representative of the target population? | 4.3. Are the measurements appropriate? | 4.4. Is the risk of nonresponse bias low? | 4.5. Is the statistical analysis appropriate to answer the research question? |
| --- | --- | --- | --- | --- | --- | --- | --- |
| Emanuel 2008 | yes | yes | yes | yes | yes | yes | yes |
| Lkhoyaali 2015 | yes | yes | yes | yes | yes | yes | yes |
| Akpan-Idiok, 2014 | yes | yes | yes | yes | yes | yes | yes |
| Akpan-idiok 2020 | yes | yes | yes | yes | yes | yes | yes |
| Onyeneho, 2021 | yes | yes | yes | yes | yes | yes | yes |
| Gabriel 2021 | yes | yes | yes | yes | yes | yes | yes |
| Muliira, 2019 | yes | yes | yes | yes | yes | yes | yes |
| Yusuf, 2011 | yes | yes | yes | yes | yes | yes | yes |
| Jite 2021 | yes | yes | yes | yes | yes | yes | yes |
| O'Neil, 2018 | yes | yes | yes | yes | yes | yes | yes |
| Kizza 2020 | yes | yes | yes | yes | yes | yes | yes |
| Alsirafy, 2021 | yes | yes | yes | yes | yes | yes | yes |
| Katende, 2017 | yes | yes | yes | yes | yes | yes | yes |
| Muriuki, 2021 | yes | yes | yes | yes | yes | yes | yes |
| Muliira, 2019 | yes | yes | yes | yes | yes | yes | yes |

**Mixed methods**

| Study | **S1. Are there clear research questions?** | **S2. Do the collected data allow to address the research questions?** | 5.1. Is there an adequate rationale for using a mixed methods design to address the research question? | 5.2. Are the different components of the study effectively integrated to answer the research question? | 5.3. Are the outputs of the integration of qualitative and quantitative components adequately interpreted? | 5.4. Are divergences and inconsistencies between quantitative and qualitative results adequately addressed? | 5.5. Do the different components of the study adhere to the quality criteria of each tradition of the methods involved? |
| --- | --- | --- | --- | --- | --- | --- | --- |
| Buyinza, 2021 | yes | yes | yes | yes | yes | yes | yes |
| Dipio 2020 | yes | yes | yes | yes | yes | yes | yes |

**Table S2**: Characteristics of included studies and summary of key findings

Qualitative studies

| **Author, year** | **Country** | **Aims** | **Study design** | **Carer demographics** | **Recruitment site/ limitations** | **Key findings** |
| --- | --- | --- | --- | --- | --- | --- |
| (Adejoh et al., 2021) | Nigeria, Uganda, and Zimbabwe | To identify the role, impact, and support of informal caregivers of patients with advanced cancer and interaction with palliative care services in Nigeria, Uganda, and Zimbabwe | Secondary analysis of interview scripts from a multi-country cross-sectional study | --n=48  --mean age 37 (sd=13.44).  --50% female, 50% male  --carer relationship: siblings 38%, son/daughter 31%, husband 15%, parent 8%, wife 8% | Primary, secondary, and tertiary cancer facilities where palliative care services were supporting the cancer patients  Limitations  -aims of primary study and secondary study different, multinational study where multiple collecting data (training given to in-country researchers) | --minimal differences between caregiver experiences amongst all 3 countries  --the role of caregiver as coordinator of care activities (buying, managing medications, organising appointments, providing transport to hospital appointments, day to day living activities)  --caregivers often had to forgo other responsibilities/ opportunities to fulfil care role which impacted their financial situation  --caregivers reported the ongoing need for emotional support for both carer and patient  --in this study, caregiver participants and patient participants were in communication with palliative care support – what was valued was easy access to medical staff (despite lack of continuity)  --where palliative care services are involved, caregivers expressed a desire for continuity of care, more flexible availability of services, transport limitations to accessing care, long distances to travel to obtain services, limited operating hours of services, language barriers |
| (Boamah Mensah et al., 2021) | Ghana | To explore the experiences (stressors and resources) of husbands as primary caregivers for their wives with advanced breast cancer | Qualitative study (interviews) | --n=15 men  --age range between 34 and 55  --husbands of women with advanced breast cancer  --all employed | -caregivers were recruited from the Oncology Unit of the Komfo Anokye Teaching Hospital (KATH) where their wives were receiving treatment  Limitations: caregivers recruited from one center – minimises generalisability of results | --marital status as strong motivator for obligations as a motivator for considering/ persisting with role of caring  --husbands considered divorce/ leaving their wives  --caregivers experienced a change in their interaction with their community due to time burden, lack of support, and stigma and secrecy associated with a cancer diagnosis  --the increased financial burden due to altered employment  --caregivers cited support from other family members, religious groups and tapping into their own spirituality, healthcare professionals (counselling, educational support) |
| (Kusi et al., 2020) | Ghana | To explore and describe the caregiving motivations and experiences among family caregivers of patients living with advanced breast cancer | Qualitative study (interviews) | --n=15  --age range 25-73 (mean 44)  --47% male, and 53% female  --86% employed  --relationship to patient: spouse 20%, child 27%, sibling 33%, friend 13% | caregivers were recruited from Komfo Anokye Teaching Hospital (KATH)  limitations  -findings on patients with advanced cancer  -caregivers recruited from one center | --very limited hospital based palliative care in Ghana, no integrative palliative care services and geographical barriers.  --caregivers had a strong sense of socio-cultural obligations to assume caring role  --Cultural protocols that associate role of carer as a woman’s domain  --role of carer as coordinator of day-to-day activities and offering emotional, and spiritual support  --spiritual leaders have a key role in illness and were invited into homes for prayer/ communion  --caregivers described a heavy financial and the limitations of the Ghanaian National health insurance scheme which meant most treatment costs are out of pocket  -caregivers described being like medical professionals at home – giving and sometimes adjusting pain medication doses, wound care, monitoring side effects of medications, management of cancer related lymphedema, being advocates for the patient  --caregiver participants describe the use of traditional approaches to deal with some symptoms such as lymphoedema |
| (Salifu et al., 2021) | Ghana | To explore the experiences of men living with prostate cancer and their caregivers about the challenges of providing informal care at home where palliative care services are limited | Qualitative study (interviews in 2 rounds) | --n=23  --relationship to patient: wife 43%, child 39%, mother 9%, sibling 9% | --caregivers were recruited from a tertiary hospital where men with prostate cancer were receiving care  Limitations: one author coding, all caregivers recruited from one center | --caregiver participants expressed great difficulty in accessing palliative care services  --caregivers expressed a strong sense of moral obligation to enter the caregiving role  --caregivers as coordinators of activities, and lack of training in performing practical skills, medication administration and titration – ‘doctors at home’  --caregiver participants expressed anxiety at the unknown/ disease progression or deterioration  --family conflicts on path of care – to undergo chemotherapy or not, or whether or not to use traditional healers/ alternative therapies  --due to poor access to health care services/ professionals, and high costs, caregivers and patients spoke about using herbal remedies which were not certified as effective, often making their problems worse, and also using a ‘trial and error’ approach  --caregiver participants expressed the importance of providing (good) food for the patients and the distress at food refusal due to nausea and vomiting  --caregiver patients expressed gratitude for help from doctors  --managing pain was distressing for the patient and the whole family, and accessing pain medication was also a challenge – hospitals experienced shortages of morphine too and sometimes families would obtain sub-standard medications |
| (Bessa et al., 2012) | Togo | To investigate caregivers of people with cancer to understand 1) their experiences 2) their problems and ways that they overcome challenges 3) the impact of caregiving on family unit | Qualitative study (interviews) | --n=17  --mean age 43 (range 30-65)  --females 82%, males 18%  --relationship to patient: siblings 29%, wives 18%, parents 18%, children 12%, relative 6% | --Caregivers were recruited via convenience sampling from 2 Lomé Teaching hospitals  Limitations:  -convenience sampling, recruitment from a limited geographical area | --the physical, psychological challenges of the caregiver role and cultural protocols which expect caregivers to not complain/ express challenges  --the challenging of navigating other roles in addition to the caregiver role – balancing work, education responsibilities (role conflict, role overload)  --the massive financial burden that caring, and the medical bills – caregivers often altered treatment plans/ medications to suit what they can afford  --caregivers emphasised the importance of good nutrition for a cancer patient and the challenge of providing what was deemed good food  --caregivers expressed the fragmentation of the family unit due to a cancer diagnosis – children dropping out of school, displacement/homelessness  --caregivers expressed the emotional burden of dealing with the patient’s anger/ frustration/ sadness  --caregivers also expressed **positive** elements – strengthening of family ties, respect earned by caring for a family member, financial provision from other family members, spousal commitment  --caregivers tapped into their own faith/ praying as a massive source of support  --caregivers expressed financial needs and continuity of care and a more equal playing field/ partnership when interacting with health **care** professionals  --caregiver participants expressed some concerns with interaction with healthcare professionals – being shouted at, lack of cooperation, feeling invisible |
| (Maree et al., 2018) | South Africa | To provide baseline data on the experiences of caregivers of cancer patients using the public health care system in South Africa | Qualitative study (interviews) | --n=20  -- 50% male, 50% female  --age range 20 -65, mean 41.7 (sd 10.9),  --relationship to patient: child 45%, friend 15%, partner/ spouse 20%, In laws 10%, other relative 10% | -caregivers were purposively sampled at an academic center in Gauteng  Limitations: participants recruited from one center which means the socioeconomic/] cultural nuances of this population are not generalisable | --caregivers expressed shock and denial at the cancer diagnosis, and some not knowing what cancer (shame, guilt, fear associated with not knowing)  --caregivers expressed a strong socio-cultural expectation to take on the role of carer  --caregivers expressed the breadth of their role: from assisting with day-to-day activities to caring out quite medical tasks (wound care, medication giving) to giving emotional support  --caregivers faced loss of jobs or employment opportunities, and stagnation/regression in their social lives due to taking on the role of caring (role conflict)  --caregivers expressed the high emotional burden and taking on negative coping techniques – sadness, pain, helplessness, isolation, substance misuse, but also relying more on God and prayer.  --“living with a changed person” – caregivers often having to deal with the anger and irritability – manipulation |
| (Githaiga, 2017) | Kenya | To explore the experiences of a group of Nairobi women caring for a family cancer patient at home | Qualitative study (interviews and focus groups) | --n=20  -- age range 33-52  --relationship to carer: spouse 14%, child 29%, sibling 29%, parent 29% | -caregivers were sampled using criterion sampling via snowball sampling, personal contacts and two sources from a local hospice | --caregivers highlighted the idea of role of reversal – children caring for parents/elders – and the cultural challenges of how children cannot be exposed to a parent’s nakedness, and the emotional burden this carries.  --married caregivers spoke about the sensitivity of navigating cultural protocols of where caring take place – use of the in-laws’ space to care for their own family members, and where their priorities/ allegiance should lie |
| (Githaiga, 2015) | Kenya | To contribute a nuanced understanding of  the family cancer caregiver role in Nairobi, Kenya | Qualitative study (interviews) | --n= 20  --all women,  --no demographic data given | -- caregivers were recruited via criterion sampling | --caregiving is viewed as a woman’s domain and seen as an ‘inherently’ woman’s role  --some caregivers expressed their reluctance of taking on the caregiver role despite assumptions/expectations laid on them  --caregivers spoke about the role of the whole community in caring for a patient but also simultaneously expressed the tensions that arose from volume of people at times involved, and the invasion of the nuclear family space |
| (Mlaba et al., 2021) | South Africa | To explore the social experiences of the families caring for members living with cancer in KwaZulu-Natal | Qualitative study (interviews) | --n=20  --ages ranged from 21 to 84 years  --70% female, 30% male  --Patient's relationship to caregiver: grandmother 10%, aunt 15%, grandson 10%, husband 15%, wife 20%, mother 20%, daughter 5%, husband 5% | -purposive sampling of caregivers who were recruited through referral by patients who attended cancer support groups and non-governmental organizations, such as hospices and homes for the sick in Kwazulu-Natal  Limitation – only one family member interviewed | --caregivers expressed a strong sense of family responsibility that spurred them on in their caring role  --caregiving was associated with positive and negative changes in relationships between the caregiver and patient – from difficulty dealing the anger from the patient to strengthened relationships through the caring process  --caregivers highlighted the idea of ‘role reversal’ – children caring for parents who were previously taking care of them.  --caregivers expressed a significant disruption in their social lives and the negative impacts on their mental health (hopelessness, lack of sleep)  --caregiving as an opportunity to deepen faith  --caregiving as a communal task – support received from friends, family, neighbours such as providing respite care or taking over tasks such as cooking and cleaning  --caregivers also highlight how isolated they are from families, and how at times, families can offer one type of support and not the other: offer financial support and not the much-needed hands on day-to-day support  --caregiving comes with a significant massive burden and financial support was cited as a support need  --other support needs suggested: transportation, education and training, healthcare workers doing home visits, counselling/ psychological support |
| (Mkandawire-Valhmu et al., 2020) | Malawi | To demonstrate how gender inequality manifests through the intersecting gendered vulnerabilities of patients and their caregivers in rural Malawi | Qualitative study (interviews) | --n=14  --all female  --age 18-29y 21%, 30-49 y 29%, 50-59y 50%  --number of children in family unit 1-3 children 35%, 4-6 children 14%, 7-9 children 35%, 10+ children 7%, unknown 7% | --caregivers of cancer patients admitted as outpatients to palliative care at a Non-Governmental Organization (NGO)-affiliated community clinic in rural Central Malawi | --end of life caregiving is gendered activity (men may be in the household but not directly involved with caring)  --girl children also can take the role of caregiver – 8/14 caregivers were under the age of 18 years, with some as young as the age of 13 (boys continue to go to school, girls often take on more tasks and end up caring for the whole family)  --caregivers described worsening poverty over the course of an illness – households headed by women tend to suffer more – marriage as a safety net  --an increase in sexual and physical violence for women involved in care activities – up to 25% described domestic violence (caregivers and patient participants), and the impact it has on the children in the family  --the persistent threat of spousal abandonment in illness and being forced to have sex is relevant for women living with cervical cancer  --the importance of providing of good food and the distress this causes caregivers in instances when it is not possible to provide food |
| (Githaiga & Swartz, 2017) | Kenya | To examine the content and contexts of family end-of-life conversations | Qualitative study (focus groups, interviews) | --n=13  --focus group 1: 5 women aged 34–52 years, all professionals working in Nairobi  --focus group 2: 3 elderly widows (65–75 years) each of whom cared for a spouse with terminal cancer. –focus group 3: 3 women aged 31–36 years who each cared for a terminally ill parent,  --focus group 4: 2 women aged 27 and 33 years who served as caregivers of siblings with terminal cancer | Caregivers were recruited via snowballing sample (sources from a local hospice, personal contacts, and referrals from participants  Limitations: retrospective accounts, small sample | --the caregiver’s role described as a midwife of death  --challenges of end-of-life conversations as culturally seen as summoning/inviting death – when they do happen – culturally unclear as to who should initiate the conversation and when it should take place  --caregivers expressed the importance of communal decision making at the end of life – DNACPR – at the end of life, the importance of communal decision making is important  --caregivers described feeling invisible and insignificant in the presence of health care professionals |
| (Potgieter & Maree, 2018) | South Africa | To describe what motivates cancer patients and their families, treated at a private cancer care center in Port Elizabeth, to undergo palliative  chemotherapy | Qualitative study (interviews) | --n=11  -- 6 husbands, 2 wives and 3 sons  --ages: range 20 to 79 years, mean age 50 years | Purposive sampling was used to recruit caregivers from a private cancer clinic in Port Elizabeth  Limitation: study in a private clinic – not generalisable to whole south African population | --caregiver and patient participants aware that the palliative chemotherapy was not a cure but were driven by the hope the treatment gave even if it was not curative  --caregivers recognised the importance of quality of life – energy levels, minimal pain, good mobility  --the caregiver role as one of moral support – maintaining a positive outlook |
| (Bates et al., 2018) | Malawi | To explore concepts of wellbeing and the contribution of palliative care to wellbeing from the perspective of patients and families affected by advanced cancer | Qualitative study (photovoice) | --n=7  --mean age 44.5, range 33-66 | --caregivers were recruited from Tiyanjane clinic where a patient was receiving palliative care for a diagnosis of advanced cancer via convenience sampling | --the role of caregiver as broad – ranging from providing day to day care to emotional and spiritual support  --families express joy in the small accomplishments -- like watching a family member walk, or do a task  --caregivers described stigma and discrimination experienced by cancer patients and their families that can lead to isolation and inability to seek help (fears of being infectious, being ‘prematurely dead’, spousal abandonment)  --caregiver and patient participants express a need for cancer public education so that people know what it is and when to seek help  --children seen as ‘courage givers’ (encouraging patient participants to eat, and being seen as a source of hope)  --caregivers described the role traditional healers play, and the tension between accessing hospital care and going to a traditional healer  --caregivers highly appreciated help received from clinic – counselling, medications,  --caregivers attribute being able to ‘live’ to the care they receive from the palliative care team – services provided in the form of medications and counselling were very impactful, and in some cases, allowed patient/ caregivers to return to work. Challenges with regards to services expressed are distance, travel costs |
| (Edwards & Greeff, 2018) | South Africa | To explore the emotional challenges of a cancer diagnosis in South Africa | Qualitative study (photovoice) | --n=286  -- The ages of patient/family contributors ranged from 8 months (represented by a parent) to 86 years of age  --48.2% male, 51.8% female | --Convenience sampling was applied to referrals from interim care homes and oncology units in 9 cities in South Africa over a 20-month period | --157 patient/family contributors lived further than 50 km from a cancer treatment center  --caregiver/ patient participants documented high levels of anxiety, and the stress and fear of not knowing what to expect  --stigma associated with a cancer diagnosis was being of lesser worth, community rejection, abandonment by spouse, denial of the diagnosis, fear of contamination by cancer  --caregivers expressed a desire to be more involved in some hospital processes – accompanying patients for chemotherapy  --a cancer diagnosis can lead to the breakdown of the family unit  --positive elements: support from friends and family, medical teams, community, using a positive attitude to cope  --massive appreciation of help from cancer organisations (Cancer Association of South Africa (CANSA) and Childhood Cancer Foundation of South Africa’s (CHOC)) in the form of counselling and interim home support (respite) |

Quantitative studies

| **Author, year** | **Country** | **Aims** | **Study design** | **Carer demographics** | **Recruitment site** | **Key findings** |
| --- | --- | --- | --- | --- | --- | --- |
| (Emanuel et al., 2008) | Uganda | Pilot study to investigate the social and financial experiences of informal caregivers | Quantitative study (survey) | --n=62  -- age 15-20y 8.1%, 21-30y 22.6% 31-40y 32.3% 41-50y 25.8 more than 51 11.3%  --gender male 55.7%, female 44.3%  --relationship to patient: parent 25.7%, child 12.9%, brother/sister 17.7%, grandparent 3.2%, spouse 17.7%, aunt 8.1%, in law 1.6%, friend 3.2% | --caregivers were recruited by convenience sampling at Hospice Uganda | -- The primary tasks performed by the caregivers: general care (37%), homemaking (23%), nursing care (20%), personal care (38%), emotional and spiritual support (22%), and financial support (17%) and help with transportation (13%)  --the two biggest worries for caregivers were the patient’s illness (55%) and financial worries (28%)  --84% of caregivers reported significant financial issues  -- 15% of caregivers had ever received any prior caregiving training and 95% say they would be interested in getting trained  --77% of caregivers say they would hire care if money were not an issue |
| (Lkhoyaali, El Haj, et al., 2015) | Morocco | To assess the social, psychological, behavioural and economic impact on patient’s family caregivers | Quantitative studies (questionnaire) | --n=150  --mean age 44 years  --majority of caregivers were married  --employed full time  --53% of elderly patients lived with their families and 56% with the children | --participants were recruited from the National Institute of Oncology in Morocco | --in this population, 34% of caregivers were clinically depressed as per DSM-IV, and 62.7% reported at least one sign of depression  --anxiety and depression overlap occurred in 79.3% of participants  --care and decisions around care tend to be communal  --78.7% of caregivers reported living beyond their financial means  --75.3% of caregivers wanted maximum care for their elderly relatives and only 10% expressed desire to limit care |
| (Akpan-Idiok & Anarado, 2014) | Nigeria | To determine the perceived burden caregivers of cancer patients | Quantitative study (cross sectional, survey) | --n=210  --37% male, 63% female  --Ages: mean age 35.9 (sd 18.1)  --relationship to patient: parent 63%, spouse 21%, sibling 10%, friend 5%, brethren 2% | --cancer caregivers of patients attending University of Calabar Teaching Hospital in Calabar, Nigeria were purposively sampled  Limitations: cross sectional study, caregivers recruited from one hospital | --of the 210 caregivers, 17.6% experienced no/trivial burden, 36.2% experienced severe burden and 46.2% experienced severe burden  --The forms of burden included - physical 43.3%, psychological 43.3%, financial 41.4% and social 46.7%  --duration of care is associated with higher levels of burden – 54.1% of caregivers in a caring relationship between 1-5 months reported no/trivial burden, 39.5% of caregivers caring between 6-10 months reported moderate burden, 56.7% of caregivers caring for 11 or more months reported severe burden  --the types of tasks carried out by caregivers included: 74% needed feeding, 77% bathing, 75% dressing, 11% grooming, 72% help using toilet 72%. 82% of patients were incontinent, and 90% needed help transferring, 93% needed supervision, 6% needed assistance taking medications  --97% of caregivers managed money/finances for the family |
| (Akpan-Idiok et al., 2020) | Nigeria | To determine the burden experienced and coping strategies among caregivers of advanced cancer patients attending University of Calabar Teaching Hospital (UCTH), Nigeria | Qualitative (cross sectional, questionnaire) | --n=210  ----37% male, 63% female  --Ages: mean age 35.9 (sd 18.1)  --relationship to patient: parent 63%, spouse 21%, sibling 10%, friend 5%, brethren 2% | --cancer caregivers of patients attending University of Calabar Teaching Hospital in Calabar, Nigeria were purposively sampled  Limitations: cross sectional study, caregivers recruited from one hospital | --coping strategies adopted by caregivers were divided into problem focused and emotion focused coping strategies  --problem focused coping strategies included: accepting the diagnosis and making life adjustments, making new priorities in life, and learning to appreciate more things in life  --emotion-focused coping: encouraging family unity, increasing self-esteem, and increasing empathy |
| (Onyeneho & Ilesanmi, 2021) | Nigeria | To assess the burden of care among family  caregivers of patients living with cancer in University  College Hospital, Ibadan. | Qualitative study (cross sectional, survey) | --n=201  -- mean age 37.68 (sd 14.29)  --female 60.4%, male 39.6%  --relationship to care receiver - brother 6.6%, sister 11%, parent 65.4% husband 9.3%, wife 7.7% | --purposive sampling of caregivers of patients admitted or attending radiotherapy clinics at University Hospital | -- 35.7% caregivers reported no burden, 44.5% reported mild burden, 15.4% reported moderate burden while 4.4% reported severe burden  --caregivers reported a significant physical burden of care which altered their own health and wellbeing (altered sleep, altered eating habits, restlessness, exhaustion)  --caregivers experienced psychological effects from caring (frustration, loss of hope, sadness, nervousness)  --caregiving experienced changes in their social life (reduced leisure time, social isolation)  -- caregiving had a significant impact on finances |
| (Gabriel et al., 2021) | Nigeria | To determine the psychological, physical, social, and spiritual needs of caregivers of cancer patients, and to assess cancer health literacy | Qualitative study (cross sectional, survey) | --n=120  --mean age 36.1 (sd 11.55)  --male 16.7%, female 83.3%  --relationship to patient: parent 2.5%, sibling 13%, spouse 18%, child 55%, friend 9.2%, other 2.5% | --caregivers were nominated by patients living with cancer undergoing treatment | --caregiver participants identified information, health care staff and psychological support needs – the highest needs were reported for family support and spiritual support needs (85% and 81.7% respectively)  --QoL was measured using City of Hope Quality of Life (Family Version) tool – caregivers had a mean score of 180 (maximum score 370) – lowest scores for physical and spiritual wellbeing  --cancer health literacy was measured using Cancer health literacy (CHLT-6) and 60.8% of caregivers were found to have limited cancer health literacy  --Cancer health literacy was negatively correlated with age and positively correlated with education |
| (Muliira & Kizza, 2019) | Uganda | To describe the extent of depression and anxiety  symptoms among family caregivers | Qualitative study (cross sectional, survey) | --n=284  --age: mean 36 (sd 13.8)  --male 26.8% female 73.2%  --caregiving hours per week less than 48 20.1%, 49-120h 32%, more than 121h 47.5%  --relationship with patient spouse 20%, child 38%, other 41% | --caregivers of cancer patients receiving care at Hospice Africa Uganda and Uganda Cancer Institute  --convenience sampling carried out  Limitations: convenience sample, self-reported outcomes, cross sectional study, one location of recruitment | --of the 284 caregivers surveyed, 35.2% had clinically significant symptoms of anxiety and 48.2% had clinically significant symptoms of depression  --from logistic regression analysis, the largest predictors of anxiety and depression are severe impact of caregiving on physical health and poor self-rated health, and severe pain levels in patient participants |
| (Yusuf et al., 2011) | Nigeria | To survey of caregivers attending clinics with their relatives | Quantitative study (cross sectional, questionnaire) | --n=103  --58% men, 42% women  --mean age 38  --72% of caregivers were sons/daughters of the patients | --caregivers of patients attending oncology outpatient clinics at Ahmadu Bello University teaching hospital | --49.5% of caregivers reported high caregiving burden, and 46.6% reported high psychological burden  --all caregivers in this study lived in a multigenerational household with majority of them having a large family with young children  --in this study, the highest burdens of care were reported by males in caregiving roles |
| (Jite et al., 2021) | Nigeria | To assess the burden of the caregiving role on the family caregivers of women with advanced breast cancer | Quantitative study (cross sectional, survey) | --n=157  -- male 53%, female 47%,  --mean age 41.6 (sd 14.7)  --relationship between family caregiver and patient: patient's: spouse 27.4%, daughter 25.5% son 14%, sister 12.1%, brother 5.7%, mother 5.1%, in-laws 4.5%, extended family 2.5%, non-family member 2.5%, father 0.6%  --intensity of caregiving (h/week) less than 7h 27.4%, 7-24h 31.8%, 25-48h 9.6%, more than 48h 30.6% | -caregivers of patient participants attending the radiation oncology clinic of the University College Hospital, Ibadan,  Nigeria  --simple random sampling | --32.5% of caregivers reported mild dysfunction in activities of daily living in their care recipients, while 12.1% reported moderate dysfunction  -- mean caregiver burden of 29.84 ± 13.9 determined by the Zarit burden interview (ZBI)  --52.2% of caregivers experienced mild burden of care while 17% and 2.5% experienced moderate and severe burdens, respectively  --older age of the caregiver was associated with higher caregiving burden  --low level of education was associated with higher caregiving burden  -- in this population, daughters of care recipients reported a higher burden of care  --in logistic regression analysis, previous hospitalisation of a care recipient and dysfunction in the care recipients ADLs were the most significant predictors of high caregiver burden |
| (O'Neil et al., 2018) | South Africa | To understand  the challenges faced by home caregivers of patients at the end of life (EoL) in South Africa | Qualitative study (survey) | --n=174  --male 27%, female 73%  --relationship to patient: spouse 34.3%, child of patient 34.3%, sibling 13.4%, parent of patient 8.1%, extended family 7.6%, non-family 2.3%, unknown 2% | --caregivers were identified by patients receiving care from Chris Hani Baragwanath Academic Hospital (CHBAH) | --Caregivers of patients dying in a facility reported greater levels of shame (OR 4.57, 95% CI 1.07 - 19.59) and sadness (OR 3.15, 95% CI 1.22 - 8.12)  --Caregivers of patients who died at home reported greater difficulty when ‘‘interacting with the patient’’ (OR 4.21, 95% CI 1.34 - 13.23), pain (OR 3.0, 95% CI 1.09 - 8.43), insomnia (OR 5.86, 95% CI 1.09 - 31.49), fatigue (OR 9.8, 95% CI 1.2 - 80.37)  --female caregiver gender is associated with higher levels of burden  --the top 3 symptoms that caregivers found the most troublesome are pain, urinary and faecal incontinence |
| (Kizza & Muliira, 2020) | Uganda | to explore the determinants of quality of life of family caregivers of cancer patients receiving care from the two main cancer centers in Uganda | Qualitative study (cross sectional, survey) | --n=284  --male 26.8% female 73.2%,  --age mean 36 (sd 13.8)  --relationship with patient: spouse 20.1%, not spouse 79.9%  --hours of caregiving a week less than 120h 52.5% more than 120h 47.5%, level of knowledge about cancer pain management high 47.9% low, 52.1% high | --caregivers were recruited were recruited while the patient was hospitalised, attending the out-patient clinic, or when at home via convenience sampling  Limitations: convenience sample, cross sectional study, two urban centers | --46.8% of caregivers reported low quality of life  --low levels of education were associated with high burdens of care, as were high levels of perceived impact of caring on physical health, and low levels of knowledge around cancer management and pain management  --knowledge of cancer pain managed, and self-rated efficacy of pain management were the most significant predictors so quality of life for family care givers |
| (Alsirafy et al., 2021) | Egypt | to determine the prevalence of caregiver burden among caregivers of patients with incurable cancer two Eastern Mediterranean countries (Egypt and Saudi Arabia) | Quantitative study (cross sectional, survey) | --n=218 (165 *Egyptian caregivers)  --age: mean 35 (IQR 29-44)  --female 58.3%, male 41.7%  --relationship to the patient: daughter 27.5%, son 24.3%, sister 13.3%, husband 10.1%, wife 6.4% brother 5.5%, other 12.8% | --Purposive-convenience sampling method was used to recruit caregivers accompanying patients attending the oncology outpatient clinic or those admitted to the oncology inpatient ward | --The mean ZBI-22 score was 23.4 (9.3) and the median (IQR) was 23 (16– 31)  -- 58.7% of caregivers had significant caregiver burden  --physical burden and the caregivers role in providing support with activities of daily living was a large predictor of caregiver burden |
| (Katende & Nakimera, 2017) | Uganda | to determine the prevalence of anxiety and depression among family carers of cancer patients at the cancer care and treatment facility in Uganda | Quantitative study (cross sectional study, survey) | --n=119  --age: mean 33.03 (sd 10.69)  --female 67.2%, male 32.8%  --relationship to patient: parent 26.9%, other 1st degree relative 41.2%, extended family 26.9% | --convenience sampling of family carers of cancer patients receiving treatment at Uganda Cancer Institute  Limitations: convenience sample, urban setting (85% of Ugandan population lives in rural areas), self-reports | --45% of caregivers had high levels of anxiety  --the type of relationship to the care recipient was a significant predictor of anxiety – the more distant the relationship, the higher the levels of anxiety  --male caregivers expressed higher levels of anxiety (46.2%) than the female caregivers (43.2%)  --26% of participants had abnormal levels of depression, with male caregivers more affected than female caregivers  --more distant relationships were associated with high levels of depression |
| (Muriuki et al., 2021) | Kenya | To assess role strain among family caregivers of adult cancer patients | Qualitative study (cross sectional, survey) | --n=255  --57.6% female, 42.4% male  --83.1% aged between 36 and  60 years  --57.3% were unemployed | -caregivers of patients attending an outpatient treatment clinic in Kenyatta National Hospital  --systematic sampling | --all caregiver participants experienced some financial strain  --80% of caregivers had other responsibilities outside caregiving and needed to adjust their personal, social and work lives to accommodate the caring role  --25.9% of caregivers reported mild strain, 44.3% moderate strain and 29.8% reported severe strain |
| (Muliira et al., 2019) | Uganda | To appraise the tasks performed and caregiver burden experienced by family caregivers during the period of hospitalisation of a cancer patient | Qualitative study (cross sectional, survey) | --n=168  --male 23%, female 76%  --age: mean 36 (sd 12.7)  --employment status employed 53.6%, unemployed 46.4%  --relationship with patient spouse 27.4%, not spouse 72.6% | --caregivers of cancer patients admitted at a national referral cancer care center in Uganda (Uganda Cancer Institute [UCI]) | --even whilst the patient participant was an inpatient, caregiver participants still played a notable role in the care --emotional support (79.8%), feeding the patient (68.5%), transporting the patient to and from other medical appointments (62.5%), making and preparing meals for the patient (55%), and giving medications (46.4%).  --75% of caregivers reported severe burden of care  --the highest level of burden were associated with duration of hospital stay, performing tasks like giving medications, level of education |

Mixed methods

| **Author, year** | **Country** | **Aims** | **Study design** | **Carer demographics** | **Recruitment site** | **Key findings** |
| --- | --- | --- | --- | --- | --- | --- |
| (Buyinza, 2018) | Uganda | To determine the psychosocial and economic effects of caring for cancer patients | -mixed methods (survey, interview) | --n=169 (survey)  --n=8 (focus groups)  --female 67.5%, male 32.5%  --Age: 15-25y 19.5%, 26-22y 14.2%, 34-41y 8.3%, 42-49y 21.3%, over 49y 36.7%.  --caring sessions: 40% cared for 1-6hrs/day, 32% cared for 6-12h/day, 28% cared for more than 12h/day  --relationship to care receiver: 33% of carers were parents of the care receiver, 18.9% were grandparents of the care receiver, 18.9% were partners of care receiver | --caregivers of patients being reviewed at Hospice Uganda for palliative care (systematic sampling) | --in this sample, 52.1% of caregivers had received training  --76.9% caregivers reported a negative change in their income/finances  --71.6% found it difficult to meet their daily expenses  --87.6% of caregivers relied on someone else to meet their daily expenses  --72.2% of caregivers stated that they felt exhausted because of their caring role (echoed in focus groups) |
| (Dipio et al., 2021) | Uganda | To establish the prevalence and factors associated with depressive symptoms among family caregivers of palliative care patients at Hospice Africa Uganda | Mixed methods (survey, interviews) | --n=161 (surveyed and interviewed)  --female 64.6%, male 36.4%  --Age: 18–28 years 31.06%, 29–39 years 27.33%, 40–50 years 22.36%, 51–60 years 19.25% | -- caregivers of patient participants receiving care from 2 Hospice Africa Uganda sites  --selected by simple random sampling | --83.78% of caregivers received monetary support to buy medications and transport to the hospital  -- there was a 46% depression prevalence in this population (compared to 17% in general Ugandan population)  --54% of caregivers had no symptoms of depression, 23% had mild symptoms of depression, 14.3% had moderate symptoms of depression, 8.7% had severe symptoms of depression  --carrying out any activities of daily living was associated with higher levels of depression  --51% of caregiver participants stated that finances were a massive challenge  -in qualitative data – caregivers acknowledged the need for good nutrition and the inability to provide food was a major stressor  -some caregivers described how their own illnesses were exacerbated by the caregiving role  --caregivers described tensions between misinformation and drive to seek help from traditional healers  --caregivers described the rise in family tensions and feeling underappreciated and isolated. |
